## [Peer Review File · Nature Communications]

Reviewers' comments:

Reviewer #1 (Remarks to the Author):

In this manuscript, the authors describe a novel way to enhance Kras signalling, the main driver of PDAC, through reduced expression of the co-activator of Ral GAP and increased Ral activity. These data are supported by very strong results and elegant gene targeting. In my opinion however, the authors should increase the quality of the data shown to formerly prove that there is no hyperactivation of MAPK and PI3K pathways as a consequence of NKIRAS decreased expression., as well as provide evidence of the stage at which NKIRAS loss of expression occur. Indeed one key result is that loss of NKIRAS prevent pancreatic regeneration without Kras expression, that appear to be independent of the other signalling pathways. I hence recommend that the following points are addressed before the acceptance of the MS.

Major points:

- p6: the text is unclear with regards to which mice are in each group: I understand that floxed mice and KO mice were mixed. If so, show that the different gene targeting lead to similar loss of NKIRAS expression;
- The figure 2B is unclear, why use Dab2 as loading control; show the data in the WT mice, as it is difficult to conclude;
- The data show an acceleration of the cancerogenesis induced by Kras mutant, at all stages. In a spontaneous model of mutated Kras-induced, what is the level of kB-Ras expression. At which stage is there a loss of expression? Finally, while it is not necessary to reach the conclusions, if the authors have data to explain how their protein expression is decreased, they should show them in this MS.
- Is there further increase of Ki67 ratio in lesions of higher grade (such as in pDKO/R)?
- For NF-kB activity, in PDAC, it is not TNF α or LPS that further increase NFkB activity. Is there also no difference in NFkB activation with stimuli that are more relevant to PDAC?
- p10 With the blots shown, I am not convinced that p44 or pAkt levels are not altered (they appear increased to me) - their kinetics could be impaired as well (peak at 5 min). Show quantifications of these blots. IHC of the signalling pathways could also be shown, as in vivo signaling kinetics are different. In early PDAC initiation, it is also shown that there is a Kras hyperactivation dependent on p110 α activity, that is necessary to MAPK activation in early stages. Is there a difference in Kras activity? Control of Suppl fig 8c is not convincing.
- MIC-1C3 is mostly known as a marker of liver duct cell. Is it a recognized marker of pancreatic duct cells? If not demonstrated, show evidence.
- For the interpretation of Sox9 expression, the increase of expression could be explained by the increase of metaplastic lesions. Does the re-expression of kB-Ras 1 & 2 lead to re-normalisation of Sox 9 expression?
- While it is not necessary to the message, if the authors have data on pancreatic cancer cells with BQU57 inhibitor they should show them in the manuscript (Figure 5).
- show quantification of independent experiments in Figure 8e, F, as I am not convinced that in this scenario, NFkB activity is not altered in DKO cells.
- Figure 7, show the level of activation of other Kras downstream effectors in vivo (PI3K and MAPK) in vivo.

Minor points:

- Figure 1B, staining is unclear, show higher magnification; explain in M&M how the secondary is revealed (AEC? DAB?);
- Figure 1C: show the number of cases in the figure;
- provide scale bars in all figures when they are missing;
- Figure 3a: show number of animals in each group in the figure;
- p8: if there is no evidence of metastasis, describe the phenotype as adenocarcinoma with invasive features;
- literature of genetic inactivation of other Kras effectors, such as PI3K α or Raf is not provided and lacks in the discussion;
- it is well known that a single injection of caerulein is not sufficient to induce lesions, comment as

to why double injection is not necessary in the authors's case.

- Demonstrate specificity of Sox9 antibody in IF, IHC.
- Fig 4 b, to what refers n=17? number of animals ? FOV? in general describe to what refers the n=x in all figures (e.g. independent experiments, independent primary cell culture from different mice?) in some figure the number of independent n are missing (e.g. Fig5 e-q), so that I can assess the reproducibility of the results.

Reviewer #2 (Remarks to the Author):

Here Beel et al investigate the contribution of kB-Ras to pancreas homeostasis and neoplastic transformation. A robust compound conditional knockout model system is produced and characterized. Productive observations are made regarding participation of kB-Ras proteins to pancreatitis, acing-to-ductal metaplasia and KRAS driven tumorigenesis. The animal studies appear to be well-executed and delivered robust observations centered on kB-Ras. Connections to Ral GTPase activity are clear, however, cause/effect relationships here are not firmly established. General recommendations are to appropriately qualify those conclusions and/or develop them further prior to publication.

Specific recommendations:

1. Figure 1C: the 50/50 split between high grade and low grade status in the kB-Ras- cohort is interesting. Is there value in examining if the low grade subclass can further inform on the author's hypothesis? I.e. might kB-Ras be disconnected from Ral GTPases here for some reason, or are completely separate mechanisms involved?
2. Have direct mechanistic connections between kB-Ras and Ral-GAPs been firmly established to the extent that they can be freely inferred in this study, or is additional evaluation warranted - perhaps at least within the pateint-derived PDAC lines?
3. Though correlates are strong, evidence for causal relationships between kB-Ras phenotypes and Ral activation rest solely on the use of BQU57. Thus they rest solely on the selectivity of that compound for the target under the conditions used. I am not entirely confident that the exposure/response relationships and target selectivity profile is sufficient to make the stated conclusions, and no evidence is presented here to enhance confidence. I recommend removing those conclusions from the manuscript, or generating evidence for them though orthogonal means (perhaps using gRNAs targeting RalA/B in the patent-derived PDAC lines for example).
4. The text can be shortened extensively with no loss of clarity.

We thank the reviewers for their time and effort to help improve our manuscript. We have carefully addressed all points raised and provide a detailed point-by-point response below.

Point-by-point response:

Reviewer #1:

In this manuscript, the authors describe a novel way to enhance Kras signalling, the main driver of PDAC, through reduced expression of the co-activator of Ral GAP and increased Ral activity. These data are supported by very strong results and elegant gene targeting. In my opinion however, the authors should increase the quality of the data shown to formerly prove that there is no hyperactivation of MAPK and PI3K pathways as a consequence of NKIRAS decreased expression., as well as provide evidence of the stage at which NKIRAS loss of expression occur. Indeed one key result is that loss of NKIRAS prevent pancreatic regeneration without Kras expression, that appear to be independent of the other signalling pathways. I hence recommend that the following points are addressed before the acceptance of the MS.

Major points:

- p6: the text is unclear with regards to which mice are in each group: I understand that floxed mice and KO mice were mixed. If so, show that the different gene targeting lead to similar loss of NKIRAS expression.

In our pDKO/R and pDKO experimental groups we indeed combined results from animals with NKIRAS2^{fl/fl} and NKIRAS2^{fl/-} genotypes as stated in the manuscript. We have strengthened the previously provided evidence for a comparable reduction of kB-Ras2 expression in these two genotypes by qPCR (Fig. 2a) by including additional animals in this analysis. Additionally, we provide analysis of kB-Ras protein loss for both of these genotypes in comparison to wildtype and 1SKO/R mice by immunoblot (new Fig. 2b).

- The figure 2B is unclear, why use Dab2 as loading control; show the data in the WT mice, as it is difficult to conclude.

We have exchanged Fig. 2b and now provide a more extensive analysis of genotypes including wildtype mice (see also our response to previous comment) and use an anti-GAPDH immunoblot as loading control.

- The data show an acceleration of the cancerogenesis induced by Kras mutant, at all stages. In a spontaneous model of mutated Kras-induced, what is the level of kB-Ras expression. At which stage is there a loss of expression? Finally, while it is not necessary to reach the conclusions, if the authors have data to explain how their protein expression is decreased, they should show them in this MS.

In response to the reviewer's suggestion, we have established and performed IHC stainings of κ B-Ras and analyzed available sections of WT/R animals of an age up to 32 weeks. Unfortunately, sections of older WT/R animals are currently not available. For the period of up to 32 weeks, we could detect a tendency of decrease of κ B-Ras expression with tumour progression and provide the data for the reviewer below. Based on these findings, we at this point hypothesize that κ B-Ras loss occurs at a later stage of tumor development in the pancreas of WT/R mice when higher-grade PanINs start to develop. However, due to the limited number of samples (only two 32 weeks-old animals that presented with invasive carcinoma) available at this point, we would prefer not to include this data in the manuscript. Instead, we have added a paragraph in the discussion referring to our preliminary findings (p. 16).

To this point, we have not elucidated the mechanisms that underlie loss of κ B-Ras expression. We had previously reported that κ B-Ras levels are lower in lung cancer cell lines expressing KRas mutants than in those expressing wildtype KRas (Oeckinghaus A et al. Cell Reports 2013), which led us to believe that Ras signaling itself triggered downregulation. However, in the TMA of PDAC patients analyzed in this study we do not see a clear correlation between κ B-Ras levels and KRas mutational status. It is thus possible that loss of κ B-Ras expression is caused by independent genetic alterations to support mutant K-Ras-driven tumorigenesis.

- Is there further increase of Ki67 ratio in lesions of higher grade (such as in pDKO/R)?

We thank the reviewer for this comment. Indeed, the literature suggests that higher-grade lesions tend to be more proliferative. We have confirmed these findings in small scale with our own sections from WT/R animals (data not shown). We therefore cannot exclude that the increase in Ki67 ratio in pDKO/R pancreata is due to the presence of more higher-grade lesions in comparison to 1SKO/R animals. We state this more clearly in the text now.

- For NF- κ B activity, in PDAC, it is not TNF α or LPS that further increase NF κ B activity. Is there also no difference in NF κ B activation with stimuli that are more relevant to PDAC?

*Following the reviewer's advice, we have investigated induction of NF- κ B activity in both murine and human PDAC cells upon stimulation with the more PDAC-relevant cytokines IL-1 α and IL-1 β and provide the results in **new Figure 4g, 5h and Supplementary Fig. 4d**. Also for these stimuli, no differences in NF- κ B induction were observed. While we agree that LPS is not a relevant stimulus for PDAC, we originally included this stimulus because we had previously observed altered NF- κ B activity in macrophages upon treatment with LPS. We thus feel that showing results with all stimuli tested is still warranted. We have stated our reasoning more clearly in the text.*

- p10 With the blots shown, I am not convinced that p44 or pAkt levels are not altered (they appear increased to me) - their kinetics could be impaired as well (peak at 5 min). Show quantifications of these blots. IHC of the signalling pathways could also be shown, as in vivo signaling kinetics are different. In early PDAC initiation, it is also shown that there is a Kras hyperactivation dependent on p110 α activity, that is necessary to MAPK activation in early stages. Is there a difference in Kras activity? Control of Suppl fig 8c is not convincing.

*We now show extended kinetics of EGF stimulation in PDCs (new **Figure 4l**) and provide respective quantification of phospho-p44/42 and phospho-Akt levels (new **Supplementary Fig. 4f**). We also included a similar analysis for the patient-derived cell line Panc1 in the manuscript (new **Figure 5i** and **Supplementary Fig. 5i**) to provide convincing evidence that MAPK and Akt signaling are not altered upon loss of κ B-Ras. In addition, we provide the results of IHC staining of sections from 1SKO/R and pDKO/R pancreata for phospho-p44/42 and phospho-Akt substrates to elucidate the activation state of these two pathways during tumour development in vivo (new **Supplementary Fig. 3b**). We observe strong phosphorylation of p44/42 in both 1SKO/R and pDKO/R ducts or ductal lesions of mice of all age groups, suggesting no difference caused by loss of κ B-Ras. Staining of phospho-Akt substrates in young mice is rather weak and intensifies during tumour development, but also appears similar between comparable lesions in 1SKO/R and pDKO/R animals.*

*In **Figures 3j and 4k** we now present evidence that K-Ras activity is comparable between 1SKO/R and pDKO/R whole pancreata as well as in the respective cell line models.*

*For **new Supplementary Figure 9e** (former Supplementary Fig. 8c) we provide improved quality of data.*

- MIC-1C3 is mostly known as a marker of liver duct cell. Is it a recognized marker of pancreatic duct cells? If not demonstrated, show evidence.

The MIC-1C3 antibody has been characterized by Dr. C. Dorrell as marking surface antigens of duct (and pan-islet) cells in the pancreas [Dorrell C. et al. Isolation of mouse pancreatic alpha, beta, duct and acinar populations with cell surface markers. Mol. Cell. Endocrinol. 338, 144-150 (2011)]. We state this more clearly in the manuscript now to avoid confusion (page 9 top and page 12). We also confirmed that pancreatic cells that

are stained with MIC-1C3 indeed express the ductal marker CK19 (**Supplementary Fig. 6b**).

- For the interpretation of Sox9 expression, the increase of expression could be explained by the increase of metaplastic lesions. Does the re-expression of κ B-Ras 1 & 2 lead to re-normalisation of Sox 9 expression?

*We agree that enhanced expression of Sox9 in acinar cells in vivo could reflect the increased occurrence of metaplastic lesions. However, our cell culture experiments strongly support a direct effect of κ B-Ras/Ral signaling on control of Sox9 expression since we can demonstrate that knockout of κ B-Ras increases Sox9 levels in cells, that can be reduced by inhibition of Ral GTPases. Following the reviewer's suggestion, we have now also included the results of re-expression of κ B-Ras, which indeed results in a normalization of Sox9 expression (new **Figure 8i**). We also included results showing the effect of Ral GTPase knockdown on transdifferentiation in both organoids as well as the AR42J cell culture model to further support the notion that Ral GTPase signaling is involved in ADM control (new **Figures 8e, l, m**).*

- While it is not necessary to the message, if the authors have data on pancreatic cancer cells with BQU57 inhibitor they should show them in the manuscript (Figure 5).

*We included data demonstrating a decrease of tumour sphere formation of pDKO/R PDCs upon BQU57 treatment (new **Figure 5c**). In addition we provide data showing a similar effect of simultaneous knockdown of RalA and RalB in these cells (new **Figure 5b**; see also response to Reviewer #2, comment 3).*

- show quantification of independent experiments in Figure 8e, F, as I am not convinced that in this scenario, NF κ B activity is not altered in DKO cells.

*We provide better quality data for **Figure 8g** (former Fig. 8f) and added a quantification of three independently performed experiments to demonstrate more convincingly that NF- κ B activity is unaltered in AR42J cells upon deletion of both κ B-Ras proteins (new **Supplementary Figure 9d**). We also provide a quantification for the Sec5 PD results (new Figure 8f, former Figure 8e) in new **Supplementary Figure 9c**.*

- Figure 7, show the level of activation of other Kras downstream effectors in vivo (PI3K and MAPK) in vivo.

*We show results of IHC stainings of phospho-p44/42 and phospho-Akt substrates after cerulein injection and during the course of regeneration in 1SKO and pDKO mice in new **Supplementary Figure 8a, b**. We detect a comparable induction of phospho-p44/42 and phospho-Akt substrate levels after cerulein injection in both genotypes. Activity of both pathways decreases during acinar regeneration. In pDKO mice we can still detect weak activity after 21 days in areas that have not regenerated. We discuss these findings in the revised manuscript accordingly.*

Minor points:

- Figure 1B, staining is unclear, show higher magnification; explain in M&M how the secondary is revealed (AEC? DAB?)

*We included a higher magnification of the stainings in **Fig. 1b**. Our methods section now states that these stainings were revealed by the DAB method.*

- Figure 1C: show the number of cases in the figure;

*We have included the case numbers in **Fig. 1c**.*

- provide scale bars in all figures when they are missing;

Scale bars are now provided in all figures where appropriate (Figures 1b, 2c, 3b, 3c, 3e, 3f, 3g, 4a, 6b, 6d, 7c, 7d, 8c, 8d and Supplementary Figures 3b, 7a, 7b, 8a, 8b).

- Figure 3a: show number of animals in each group in the figure;

We now show the number of animals in each group in the figure.

- p8: if there is no evidence of metastasis, describe the phenotype as adenocarcinoma with invasive features;

According to the reviewers suggestion we have rephrased the respective passages on pages 7/8.

- literature of genetic inactivation of other Kras effectors, such as PI3Ka or Raf is not provided and lacks in the discussion;

We have included a brief summary of relevant findings on the effects of inactivation of other Ras effectors in the introduction and discussion parts and hope to now provide a broader picture of the current knowledge in the field.

- it is well known that a single injection of caerulein is not sufficient to induce lesions, comment as to why double injection is not necessary in the authors's case.

We decided to use a one-day injection protocol (7 hourly injections), which has been used previously to induce acute pancreatitis (see e.g. Halbrook CJ et al. Cell Mol Gastroenterol Hepatol 2017), to trigger a relatively mild induction as we expected to see increased effects in pDKO mice. This protocol was also preferable in respect to animal welfare considerations. As demonstrated in our study the procedure indeed was sufficient to induce ADM on the C57BL/6J background and allowed observation of regeneration processes but – as the reviewer pointed out – was not sufficient to induce lesions in wildtype animals.

- Demonstrate specificity of Sox9 antibody in IF, IHC.

*Specificity of the Sox9 antibody is now shown in IF of murine PDCs after stable knockdown of Sox9 using three different shRNAs (new **Supplementary Figures 6c and d**).*

- Fig 4 b, to what refers n=17? number of animals ? FOV? in general describe to what refers the n=x in all figures (e.g. independent experiments, independent primary cell culture from different mice?) in some figure the number of independent n are missing (e.g. Fig5 e-q), so that I can assess the reproducibility of the results.

In Fig. 4b n=17 indeed refers to 17 fields of view taken from a total of three mice. In the respective legend as well as in all other legends we have now included more detailed information on the definition of n numbers, where they do not represent independently performed experiments. We included a Statistics paragraph in the methods section that states that all n numbers that are not specified represent independently performed experiments. We also ensured that n numbers are present in all legends.

Reviewer #2:

Here Beel et al investigate the contribution of kB-Ras to pancreas homeostasis and neoplastic transformation. A robust compound conditional knockout model system is produced and characterized. Productive observations are made regarding participation of kB-Ras proteins to pancreatitis, acing-to-ductal metaplasia and KRAS driven tumorigenesis. The animal studies appear to be well-executed and delivered robust observations centered on kB-Ras. Connections to Ral GTPase activity are clear, however, cause/effect relationships here are not firmly established. General recommendations are to appropriately qualify those conclusions and/or develop them further prior to publication.

Specific recommendations:

1. Figure 1C: the 50/50 split between high grade and low grade status in the kB-Ras- cohort is interesting. Is there value in examining if the low grade subclass can further inform on the author's hypothesis? I.e. might kB-Ras be disconnected from Ral GTPases here for some reason, or are completely separate mechanisms involved?

We agree with the reviewer that it is interesting to further analyze the role of kB-Ras in the subgroup of low-grade tumors. However, additional analysis revealed that in the group of low-grade tumors, there was no significant association between KRAS mutation status and kB-Ras expression ($p=1.0$, Fisher's exact test; data not shown), the most likely molecular connection at this point. It has to be noted that PDAC is a genetically heterogeneous disease and may harbor additional molecular alterations in addition to

KRAS mutations (TP53, SMAD4, CDKN2A, ARID1A and ROBO2 mutations; ERBB2, MET, FGFR1, CDK6, PIK3R3 and PIK3CA amplifications; and inactivation of BRCA1, BRCA2 or PALB2; Waddell N, Pajic M, Patch AM, et al. Whole genomes redefine the mutational landscape of pancreatic cancer. Nature. 2015;518:495–501). It is well conceivable that κ B-Ras expression in this group might be associated with distinct molecular subgroups, as it has also been shown that histomorphology predicts molecular subtype in PDAC (N Kalimuthu S, Wilson GW, Grant RC, et al. Morphological classification of pancreatic ductal adenocarcinoma that predicts molecular subtypes and correlates with clinical outcome. Gut 2020;69:317-328). However, since the overall number of low-grade tumours in the present study (n=38) precludes an in-depth analysis of (molecular) subgroups, we feel like such correlation lies beyond the scope of the present study and should be addressed in further investigations.

2. Have direct mechanistic connections between κ B-Ras and Ral-GAPs been firmly established to the extent that they can be freely inferred in this study, or is additional evaluation warranted - perhaps at least within the patient-derived PDAC lines?

*We have included additional experiments in our manuscript demonstrating that κ B-Ras deficiency upregulates Ral-GTP level through loss of Ral-GAP function in pancreatic cells. 1.) We demonstrate that in both murine and human PDAC cells knockout of κ B-Ras leads to enhanced GTP levels of the fast-cycling RalA mutant F39L (new **Figures 4j and 5j**). This mutant has previously been shown to be regulated independently of GEF activity but to be still responsive to GAP function. Increased levels of RalA F39L-GTP in pDKO compared to 1SKO control cells thus suggests that this increase is mediated through GAP regulation. 2.) We show that a dominant-negative Ral mutant S28N, that has previously been shown to inhibit GEF-mediated Ral activation, is not able to reduce the high Ral-GTP level in murine or human κ B-Ras DKO PDAC cells (**Supplementary Figs. 4e and 5j**). This demonstrates that Ral-GTP level in pDKO cells are not enhanced via Ral-GEF-dependent signaling. Together these findings demonstrate that κ B-Ras deficiency leads to a loss of GAP function resulting in enhanced Ral-GTP levels. More detailed studies on the molecular mechanism of Ral-GAP regulation by κ B-Ras are ongoing addressing potential direct regulation of catalytic activity through conformation changes or change of subcellular localization or phosphorylation of Ral-GAP proteins through κ B-Ras binding.*

3. Though correlates are strong, evidence for causal relationships between κ B-Ras phenotypes and Ral activation rest solely on the use of BQU57. Thus they rest solely on the selectivity of that compound for the target under the conditions used. I am not entirely confident that the exposure/response relationships and target selectivity profile is sufficient to make the stated conclusions, and no evidence is presented here to enhance confidence. I recommend removing those conclusions from the manuscript, or generating evidence for them through orthogonal means (perhaps using gRNAs targeting RalA/B in the patient-derived PDAC lines for example).

We agree with the reviewer's opinion that including orthogonal evidence for the role of Ral GTPases in the described phenotypes of κ B-Ras knockout would strongly improve our manuscript, especially for the experiments related to ADM. We therefore now include

data on the effects of shRNA-mediated simultaneous knockdown of RalA and RalB for all observed phenotypes and provide the following results:

- *shRNA-mediated knockdown of Ral GTPases decreases tumor sphere formation of murine pDKO/R PDCs and the patient-derived cell line Panc1 (new **Figure 5b and k**). We have also added results for the use of BQU57 in this system (new **Figure 5c**).*
- *Formation of duct-like structures by primary pDKO acini is reduced by knockdown of Ral GTPases in our organoid model (new **Figure 8e**)*
- *Knockdown of Ral GTPases impairs the upregulation of Sox9 in WT AR42J cells upon stimulation as well as the generally enhanced Sox9 levels in AR42J DKO cells demonstrating the importance of Ral for regulation of Sox9 expression (new **Figure 8l, m**).*

We feel that with these additional experiments we provide convincing evidence to support a role of Ral signaling in the observed phenotypes.

4. The text can be shortened extensively with no loss of clarity.

In response to the reviewer's comment, we have shortened the text.

REVIEWERS' COMMENTS:

Reviewer #1 (Remarks to the Author):

The authors have replied to all the questions in a convincing manner and the message is now clearer: the positioning (with regards to current knowledge in the field) of this novel downstream effector of Kras in pancreatic cancerogenesis is important.

There is however one staining I do not clearly understand : Figure 7 amylase staining DKONaCl 48h and 21d - why is it not stronger in acini?

Reviewer #2 (Remarks to the Author):

Revisions are acceptable

Editorial Office

Datum 08.06.2020
Robert-Koch-Straße 43
48149 Münster
www.imtb.uni-muenster.de

Dr. Andrea Oeckinghaus.

Tel. +49 (0) 251 83-51606
Fax +49 (0) 251 83-55303
oeckinga@ukmuenster.de

Submission of revised manuscript NCOMMS-19-36233A
Response to reviewers comments

Please find below our point-by-point response to the reviewers comments.

Reviewer #1 (Remarks to the Author):

The authors have replied to all the questions in a convincing manner and the message is now clearer: the positioning (with regards to current knowledge in the field) of this novel downstream effector of Kras in pancreatic cancerogenesis is important.

There is however one staining I do not clearly understand: Figure 7 amylase staining DKONaCl 48h and 21d - why is it not stronger in acini?

Both NaCl panels showed that our amylase staining is specific since acinar cells but not Langerhans islets or ductal structures are stained by the antibody. So the staining is stronger in acinar cells than other pancreatic cell types.

We thus assume that the reviewer wondered why the staining in acini seemed less intense under NaCl than Cerulein conditions (1SKO) especially after 48h hours. Based on the reviewer's comment we have carefully revisited all our amylase stainings and can confirm that there really is no difference in staining intensity between these conditions. We therefore now provide better quality data that clearly demonstrates this fact (Figure 7c and d).

Reviewer #2 (Remarks to the Author):

Revisions are acceptable

We hope to have clarified the remaining issue to your satisfaction.

Best regards,

Andrea Oeckinghaus