# Supplementary material for: κB-Ras and Ral GTPases regulate acinar to ductal metaplasia during pancreatic adenocarcinoma development and pancreatitis
Source: Nat Commun. 2020 Jul 8;11:3409. doi: 10.1038/s41467-020-17226-0 (PMC7343838; doi:10.1038/s41467-020-17226-0)
Supplement: Supplementary file 1 — Supplementary Information [file 41467_2020_17226_MOESM1_ESM.pdf]

κB-Ras and Ral GTPases regulate acinar to ductal metaplasia during pancreatic adenocarcinoma development and pancreatitis

Beel et al.

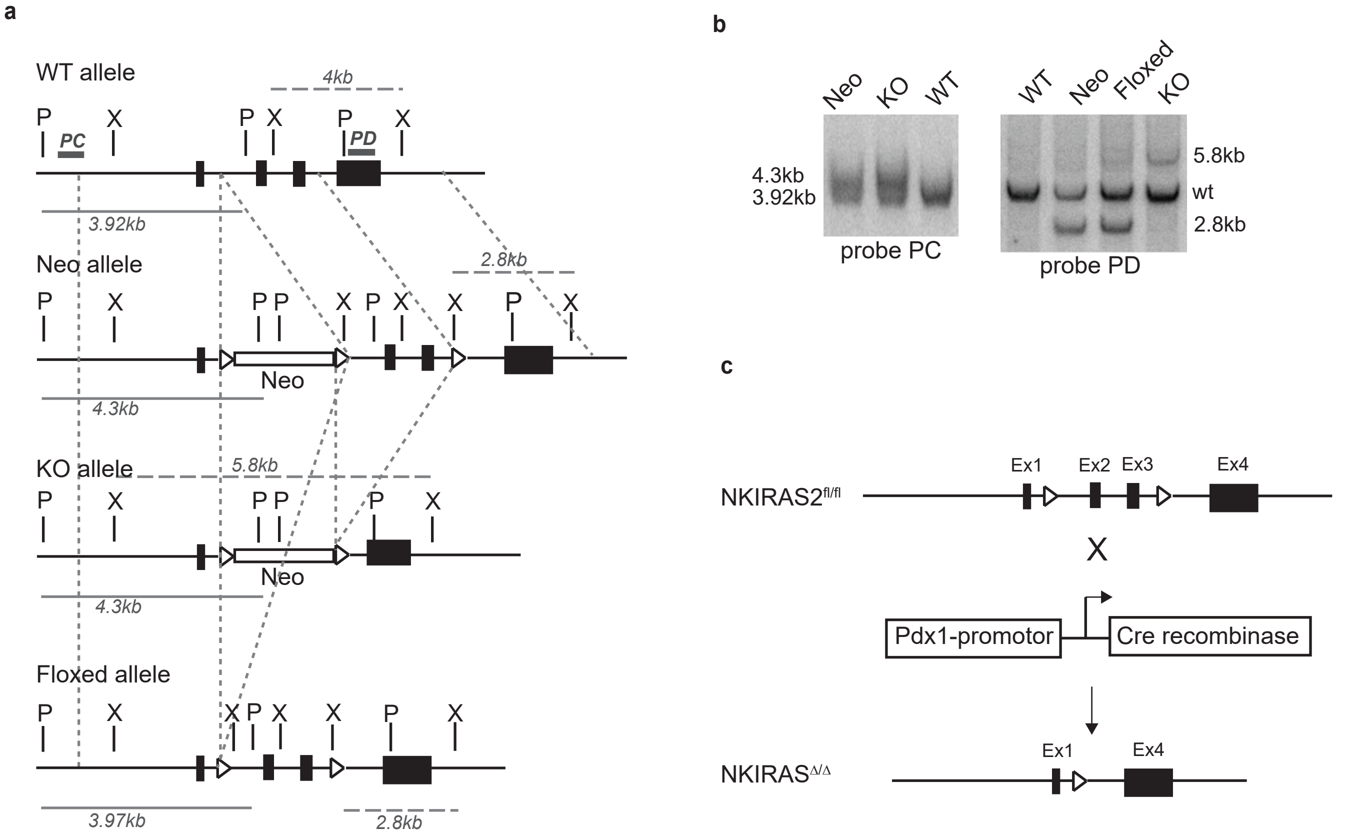

Supplementary Figure 1: Generation of conditional κB-Ras2 knockout mice.
a, Top: Schematic representation of the targeted κB-Ras2 locus. open triangles, loxP sites; neo, neomycin expression cassette; black boxes, exons 1 to 4; grey boxes, probes PC and PD Southern blotting; P, PstI; X, XbaI. The fragments and their sizes expected in Southern blotting are indicated by dashed lines.
b, Southern blotting results of the four different alleles of κB-Ras2 using PC and PD probes.
c, Schematic of Pdx1-Cre recombinase driven conditional κB-Ras2 deletion.
Data are provided as a Source data file.

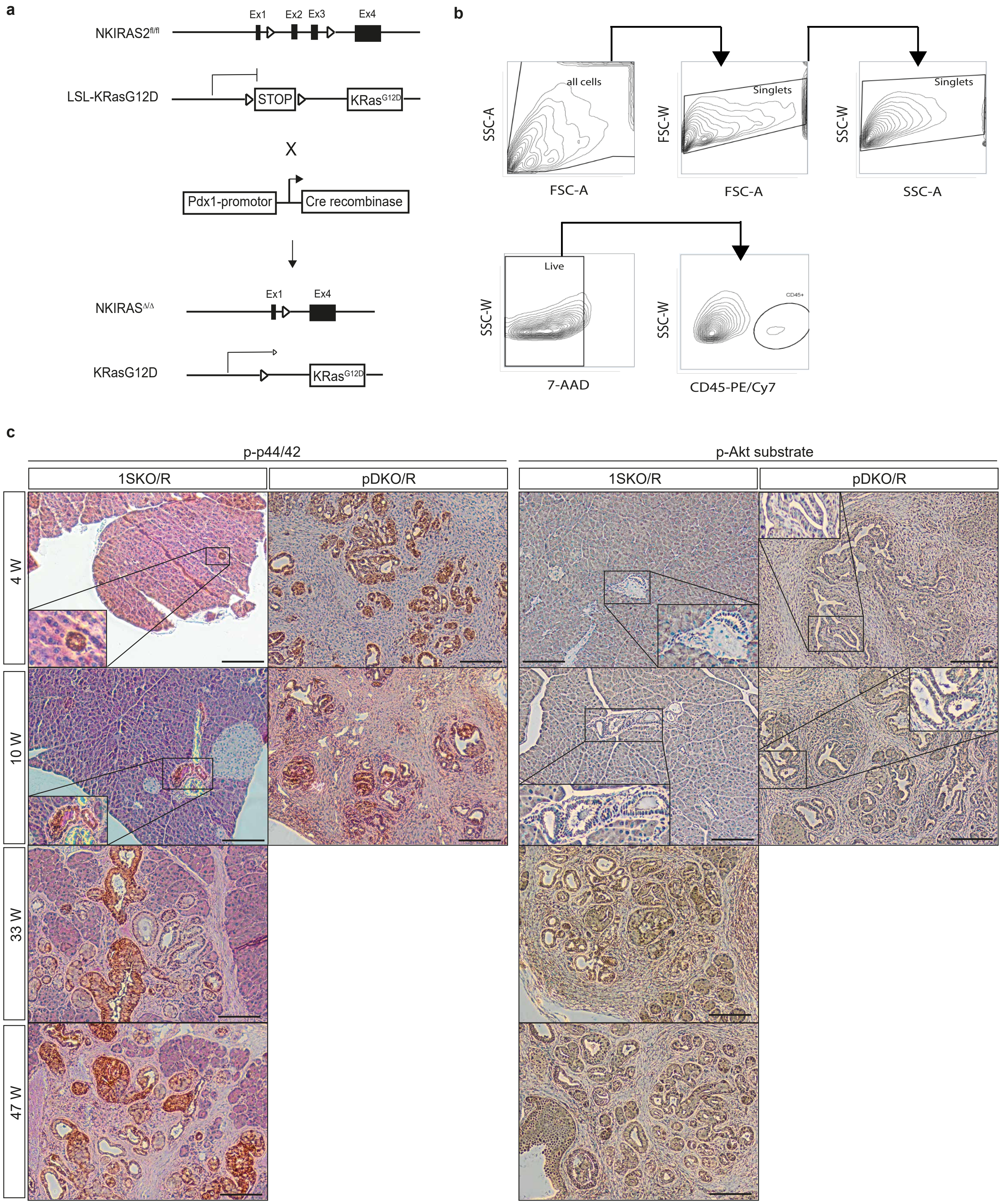

Supplementary Figure 2: κB-Ras deficiency cooperates with oncogenic K-Ras to promote invasive pancreatic adenocarcinoma.

a, Schematic of Pdx1-Cre recombinase driven expression of KRasG12D from the LSL-KRASG12D allele and simultaneous deletion of conditional κB-Ras2. **b**, Gating strategy for detection of CD45-positive cells in pancreata by FACS (see Figure 3g). **c**, Paraffin sections of pancreata from 4, 10, 33 and 47 weeks-old mice with the indicated genotypes were stained with antibodies against phospho-p44/42 or phospho-Akt substrate. Scale bars: 40 μm.

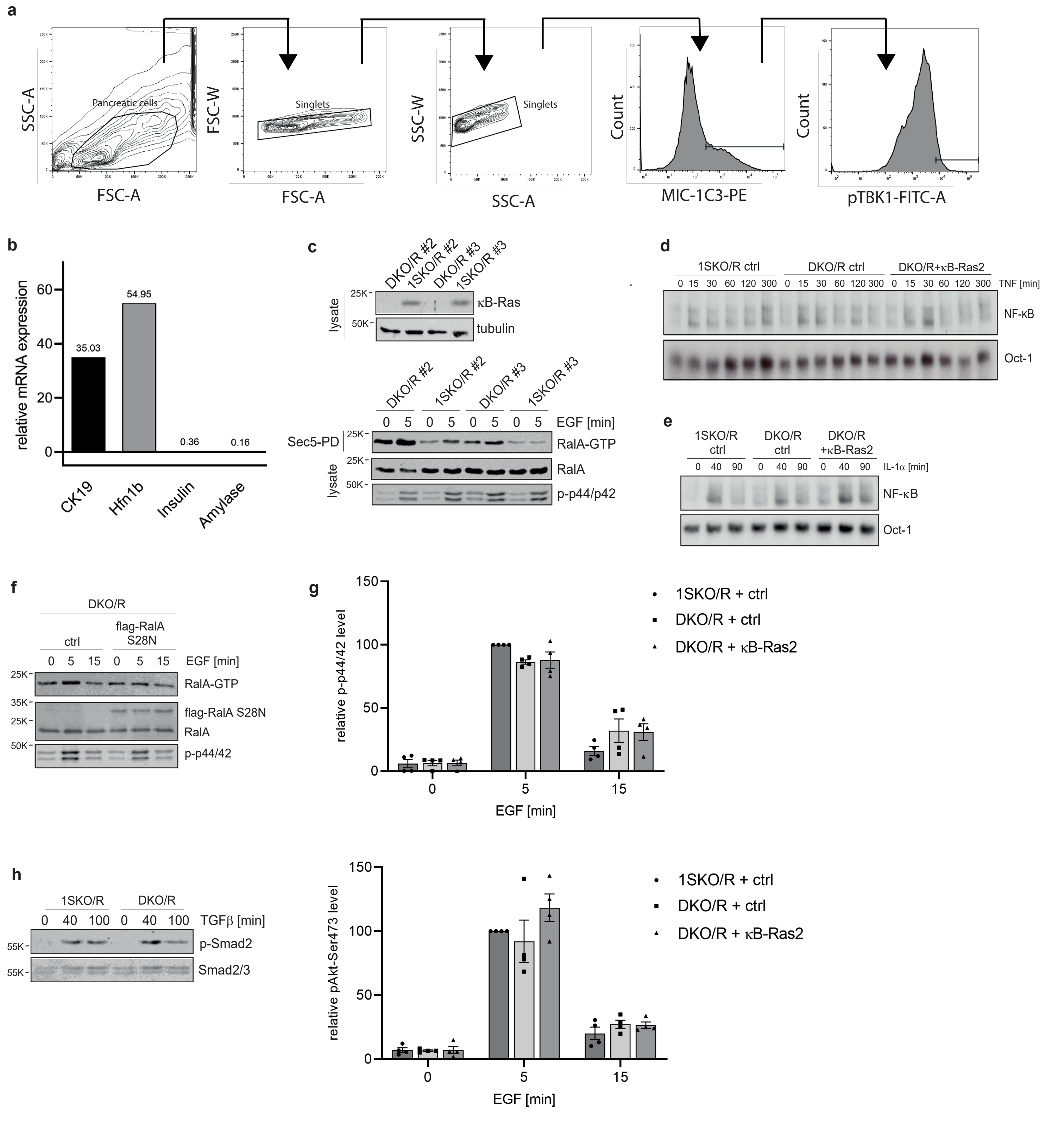

Supplementary Figure 3: κB-Ras proteins control Ral activity in pancreatic ductal tumor cells.
a, Gating strategy for analysis of phosphorylation of TBK1 and p65 in primary pancreatic ductal cells (see Figure 4c, d). **b**, RT-qPCR analysis of different pancreatic cell type markers (CK19, Hnf1b: ductal cells; insulin: islet cells; amylase1: acinar cells) of pDKO/R PDCs. Values are normalized to GAPDH and given relative to the cell fraction separated upon initial differential trypsinization set as 1. n=1. **c**, Expression level of κB-Ras total protein in derived PDC cell lines of the indicated genotypes was analyzed by immunoblot. RalA-GTP level were analyzed in two additional, independently derived pairs of PDCs by GST-Sec5 pulldown and immunoblot. **d**, EMSA using whole cell extracts from TNFα (10 ng/ml) stimulated 1SKO/R ctrl, DKO/R ctrl and DKO/R+κB-Ras2 cells and the indicated probes. **e**, EMSA using whole cell extracts from IL-1α (10 ng/ml) stimulated 1SKO/R ctrl, DKO/R ctrl and DKO/R+κB-Ras2 cells and the indicated probes. **f**, DKO/R PDCs were either transfected with empty plasmid or a Flag-RalA S28N construct, stimulated with EGF (100 ng/ml), lysed and subjected to GST-Sec5 pulldown. Endogenous RalA-GTP levels were analysed by immunoblot. **g**, Quantification of phospho-p44/42 and phospho-Akt Ser473 levels after EGF stimulation (100 ng/ml) in 1SKO/R ctrl, DKO/R ctrl and DKO/R + κB-Ras2 PDCs. Data are presented as mean values +/- SD. n=4 independent experiments. **h**, 1SKO/R and DKO/R PDCs were stimulated with TGFβ and phospho-Smad2 level analysed by immunoblot. Source data are provided as a Source data file.

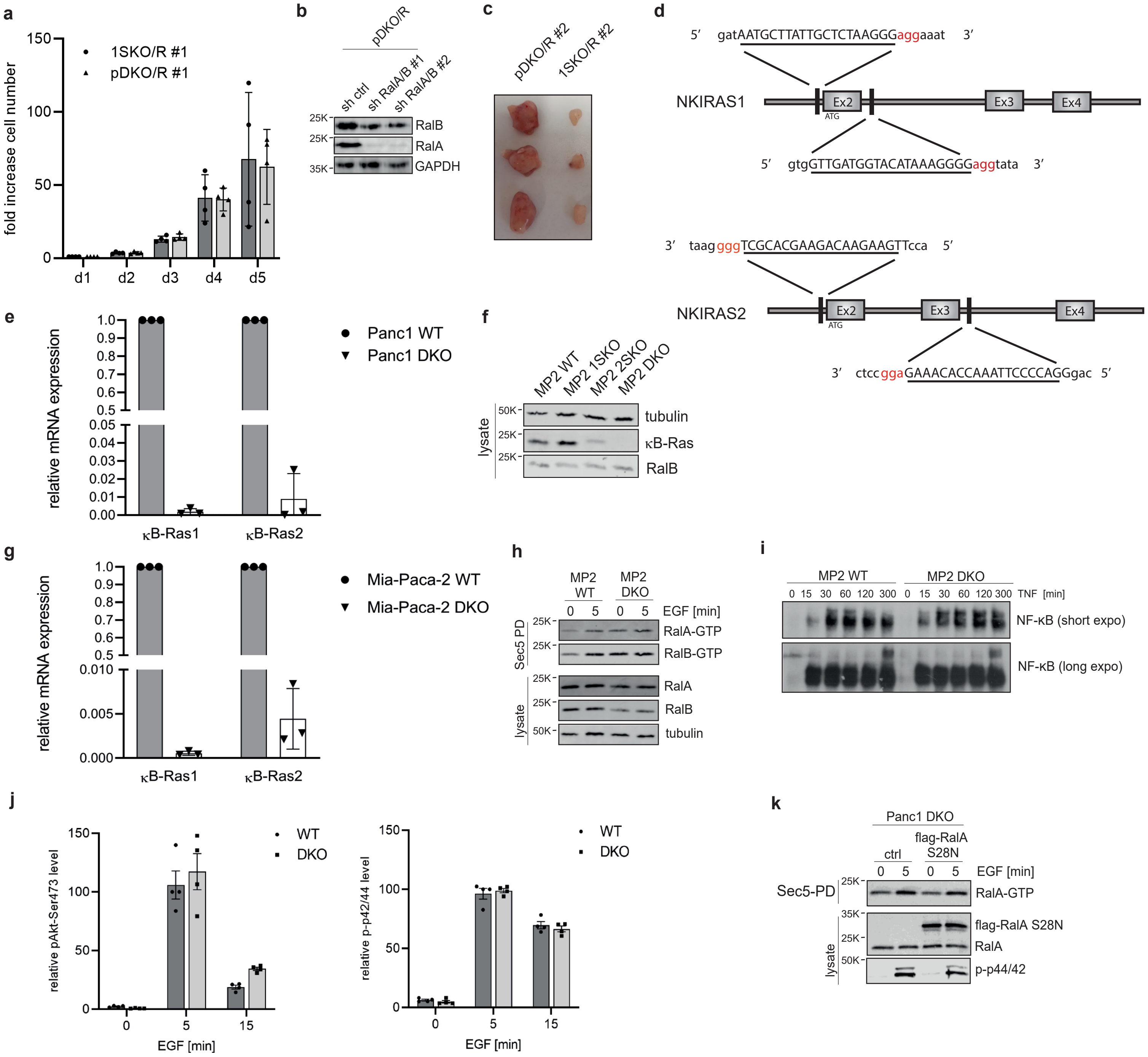

Supplementary Figure 4: κB-Ras deficiency alters proliferation characteristics of PDCs.
a, 1SKO/R and pDKO/R adherent cell proliferation analysed over five days via Cell Titer Glo Assay. Data are presented as mean values +/- SD. n=4. **b**, DKO/R PDCs cells were lentivirally transduced with either ctrl or RalA/B shRNAs and Ral protein levels analysed by immunoblot. **c**, Exemplary pictures of resected tumours grown after subcutaneous grafting of 1SKO/R and DKO/R PDCs. **d**, CRISPR/Cas9-mediated knockout strategy for human κB-Ras1 and κB-Ras2. **e**, RT-qPCR analysis of κB-Ras1 and κB-Ras2 mRNA levels after CRISPR/Cas9-mediated double knockout in Panc1 cells. Data are presented as mean values +/- SD. n=1 cells examined over 3 independent experiments. **f**, Immunoblot demonstrating levels of total κB-Ras protein in Mia-Paca2 WT, κB-Ras1 single knockout (1SKO), κB-Ras2 single knockout (2SKO) and double knockout (DKO) cells. **g**, RT-qPCR analysis of κB-Ras1 and κB-Ras2 mRNA levels after CRISPR/Cas9-mediated double knockout in Mia-Paca-2 cells. Data are presented as mean values +/- SD. n=1 cells examined over 3 independent experiments. **h**, Mia-Paca-2 WT and DKO cells were stimulated with EGF (100 ng/ml), lysed and subjected to GST-Sec5 pulldown. Ral-GTP levels were analyzed by immunoblot. **i**, EMSA using whole cell extracts from TNF-stimulated (20 ng/ml) Mia-Paca-2 WT and DKO cells. **j**, Quantification of phospho-Akt Ser473 and phospho-p44/42 levels in Panc1 WT and DKO cells after stimulation with EGF (100 ng/ml). Data are presented as mean values +/- SEM. n=4 independently performed experiments. **k**, Panc1 DKO cells were transfected with either empty plasmid or Flag-RalA S28N, stimulated with EGF (100ng/ml), lysed and subjected to GST-Sec5 pulldown. Endogenous RalA-GTP were analysed by immunoblot.
Source data are provided as a Source data file.

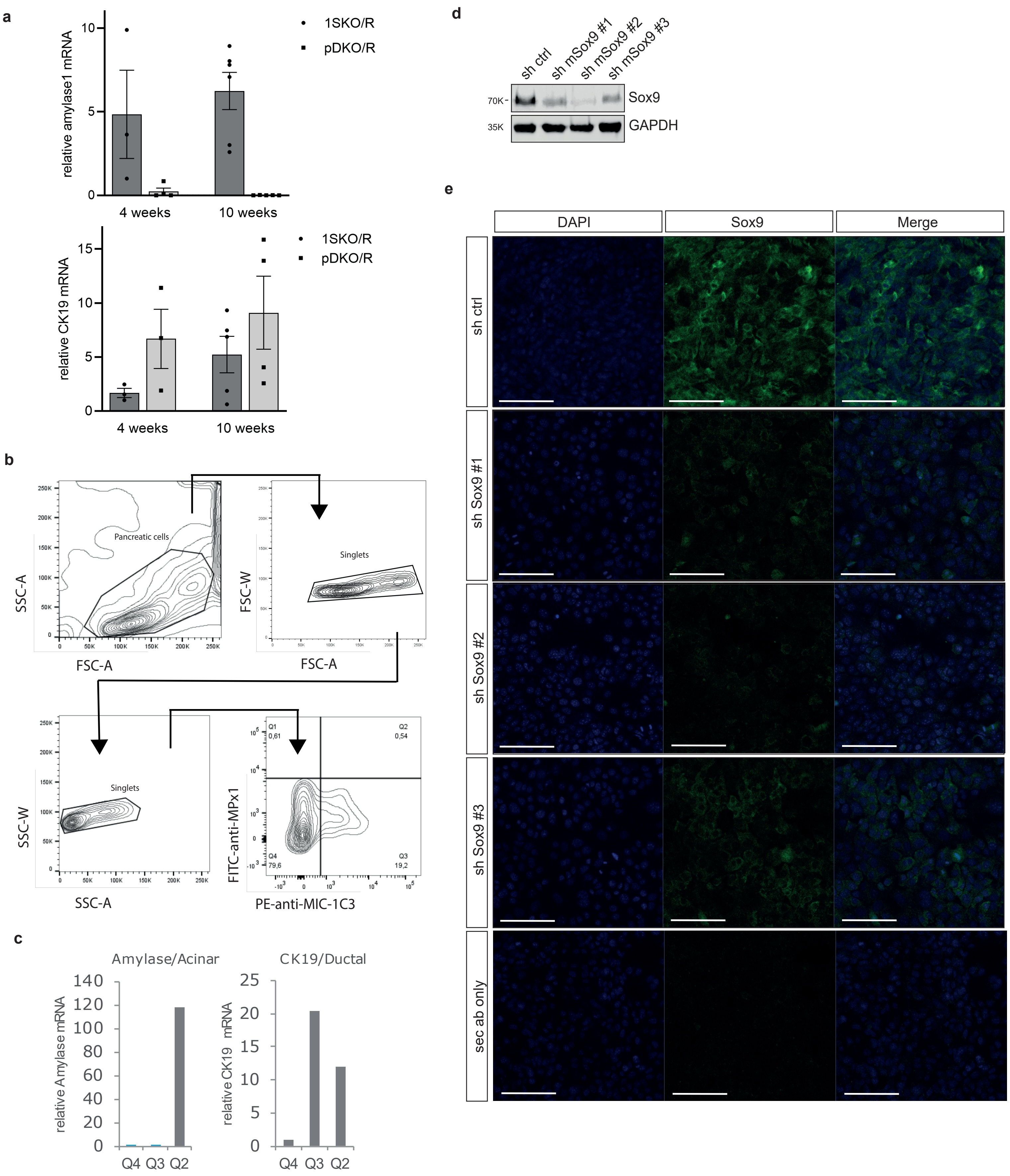

Supplementary Figure 5: Regulation of ADM in the K-Ras^{G12D}-expressing pancreas by κB-Ras proteins.
a, RT-qPCR analysis of amylase1 and cytokeratin19 mRNA levels in whole pancreatic tissue from 1SKO/R and pDKO/R animals at 4 and 10 weeks of age normalized to actin levels. Data are presented as mean values +/- SD. Amylase1: 4 weeks: n=3. 10 weeks: n=6; cytokeratin 19: 4 weeks: n=3. 10 weeks: n=4 (samples from individual mice). **b**, Gating strategy for analysis of primary pancreatic cell populations (see Figure 6c). **c**, RT-qPCR analysis of marker mRNA expression of the indicated gates used in FACS analysis (see Figure 6c). n=1. **d**, DKO/R PDCs were stably transduced with either ctrl or Sox9 shRNAs. Protein lysates were analysed for Sox9 expression.
e, DKO/R PDCs with Sox9 knockdown were fixed, stained for Sox9 and analyzed by fluorescence microscopy. Scale bars: 100 µm.
Source data are provided as a Source data file.

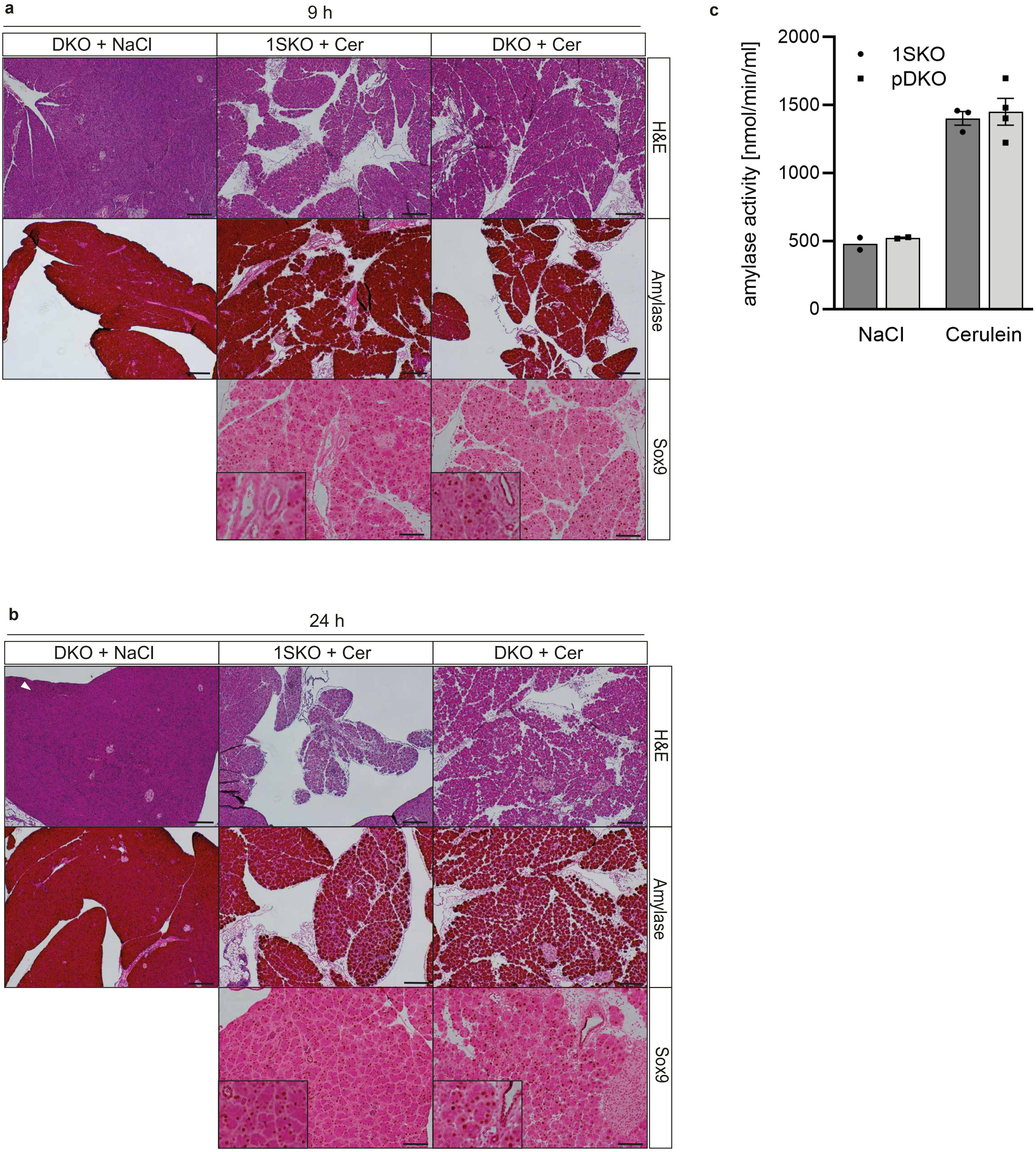

Supplementary Figure 6: κB-Ras deficiency impairs acinar regeneration after acute pancreatitis.
a and **b**, Paraffin sections of pancreata from mice with the indicated genotypes 9 h (**a**) and 24 h (**b**) after cerulein or saline injection were stained with hematoxylin/eosin or antibodies against amylase1 or Sox9. hematoxylin/eosin and amylase scale bars: 50 µm. Sox9 scale bars: 100 µm. **c**, Serum was prepared from animals sacrificed 9 h after the first cerulein injection and analyzed for amylase levels. Data are presented as mean values +/- SD. NaCl-injected animals: n=2, Cerulein-injected animals: 1SKO n=3, DKO n=4 (individual animals analysed). Source data are provided as a Source data file.

a

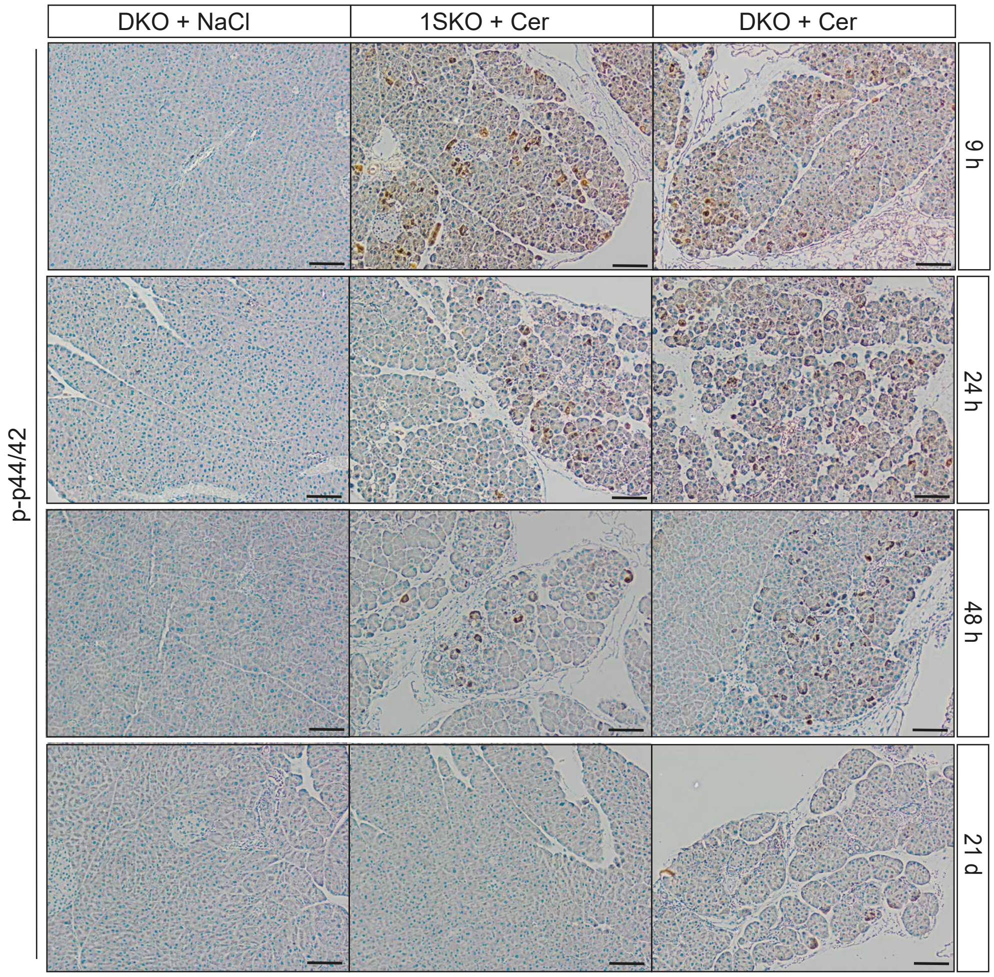

b

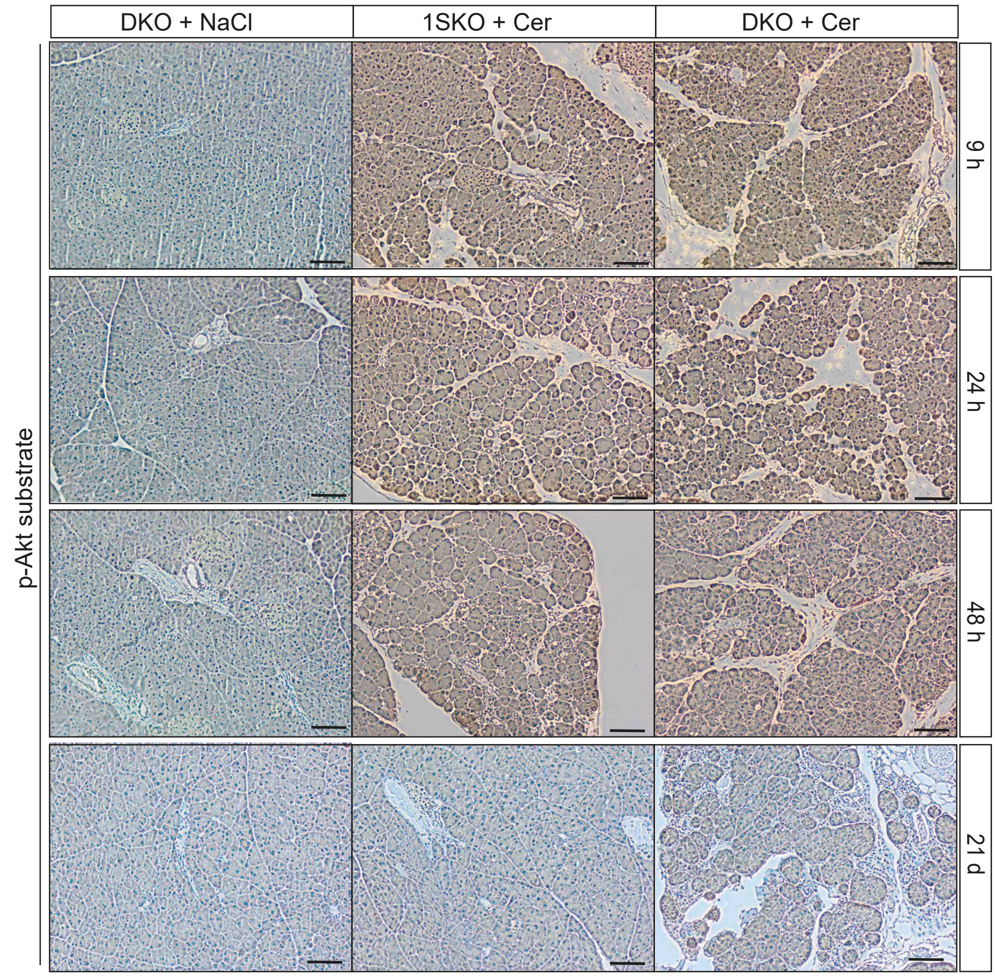

Supplementary Figure 7: κB-Ras deficiency does not affect Erk and Akt signaling after cerulein injection.
a and **b**, Paraffin sections of pancreata from mice with the indicated genotypes 9 h, 24 h, 48 h and 21 d after cerulein or saline injection were stained with antibodies against phospho-p44/42 (**a**) or phospho-Akt substrate (**b**).
Scale bars: 50 µm.

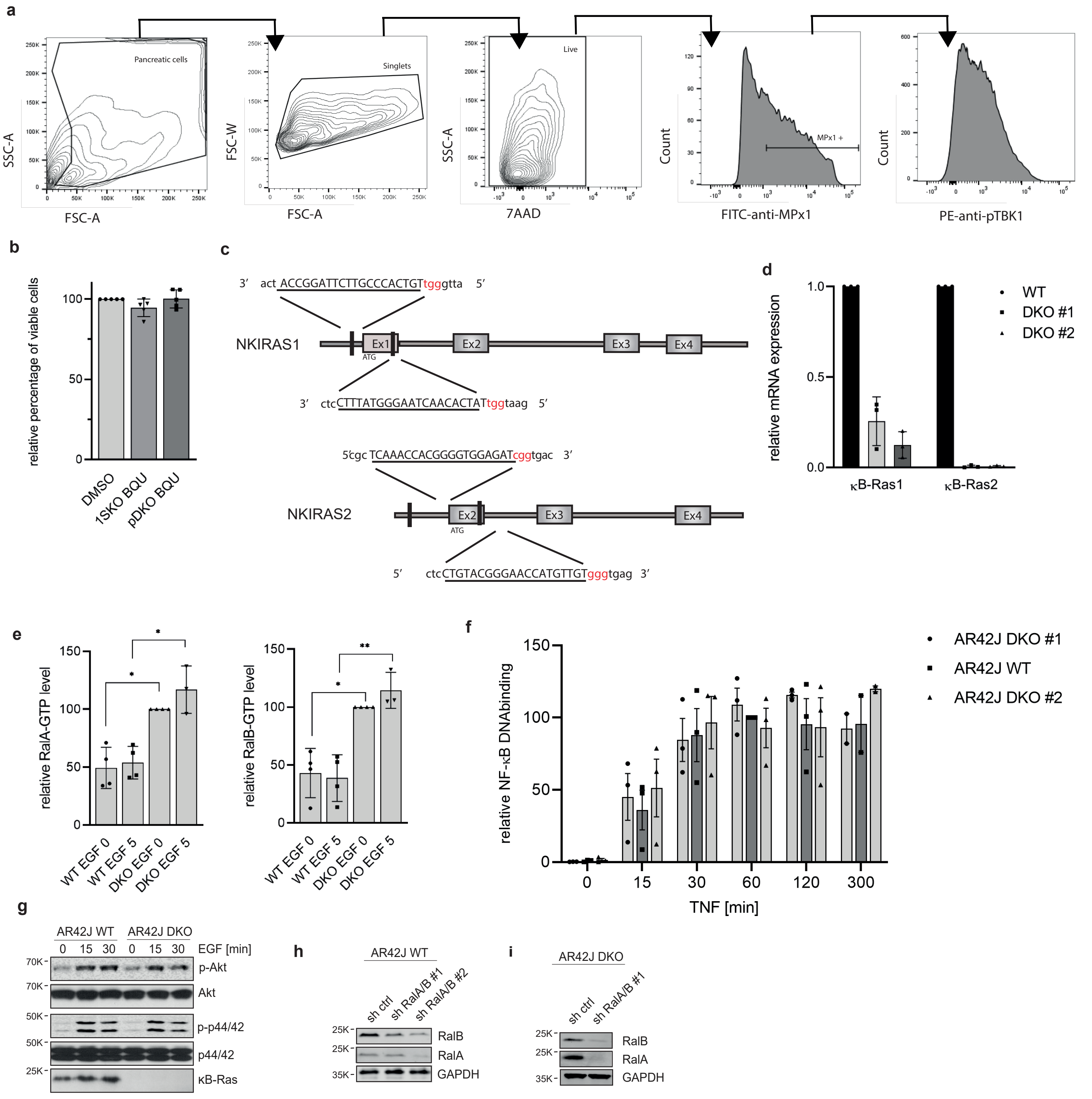

Supplementary Figure 8: κB-Ras deficiency enhances acinar Ral activity and ADM.
a, Gating strategy for FACS analysis of signaling events in primary pancreatic acinar cells (see Figure 8a). **b**, Cell viability analysis of collagen-embedded acini. Data are presented as mean values +/- SD. n=5 independently performed experiments. **c**, CRISPR/Cas9-mediated knockout strategy for rat κB-Ras1 and κB-Ras2. **d**, RT-qPCR analysis of κB-Ras1 and κB-Ras2 mRNA level in wildtype and two AR42J κB-Ras DKO cell lines. Data are presented as mean values +/- SD. n=1 cells examined over 3 independent experiments. **e**, Quantification of RalA-GTP and RalB-GTP levels after GST-Sec5 pulldown in AR42J WT and DKO cells stimulated with EGF (100 ng/ml). Data are presented as mean values +/- SD. n=4 (independently performed experiments). RalA-GTP: * p=0.0108 (unstimulated); * p=0.015 (stimulated). RalB-GTP: * p=0.0127 ** p=0.0026. Welch's t-test, two-tailed. **f**, Quantification of EMSA using whole cell lysates of TNFα (20 ng/ml) stimulated AR42J WT and two AR42J κB-Ras DKO cell lines. Data are presented as mean values +/- SEM. n = 3 (independently performed experiments). **g**, AR42J WT and DKO cells were stimulated with EGF (100 ng/ml), lysed and levels of phospho-Akt Ser473 and-phospho-44/42 analysed by immunoblot. **h**, AR42J WT cells were lentivirally transduced with either ctrl or RalA/B shRNAs to achieve stable knockdown of RalA and RalB. Protein lysates were analysed by immunoblot for total RalA and RalB levels. **i**, AR42J DKO cells were lentivirally transduced to stably knockdown RalA and RalB. Protein lysates were analysed by immunoblot for total RalA and RalB levels. Source data are provided as a Source data file.

	N (%)
No of patients	61
No of samples	81
Age (years)	
Median	65
range	39-84
Sex	
Male	40 (65.6)
Female	21 (34.4)
Histologic grade (n=67)	
Well-differentiated/G1	0
Moderately differentiated/G2	38 (56.7)
Poorly differentiated/G3	29 (43.3)
UICC stage (n=43)	
IA	0
IB	1 (2.3)
IIA	8 (18.6)
IIB	13 (30.2)
III	7 (16.3)
IV	14 (32.6)
Tissue of origin (n=81)	
Primary tumor	35 (43.2)
Metastasis	46 (56.8)
Smoking habits (n=40)	
Smoker	8 (20)
Non-smoker/never smoked	32 (80)
Diabetes (n=42)	
Diabetic	17 (40.5)
Non-diabetic	25 (59.5)
Adjuvant CTx (Gemcitabine; n=44)	
Yes	21 (47.7)
No	23 (52.3)
Palliative CTx (n=61)	
Yes	53 (86.9)
No	8 (13.1)
Palliative RTx (n=45)	
Yes	6 (13.3)
No	39 (86.7)
KRAS codon 12/13 mutation (n=56)	
Yes	32 (57.1)
No	24 (42.9)
κB-Ras expression in tumor cells (n=56)	
Absent	17 (30)
Weak/moderate	22 (39.1)
Strong	17 (30)

Supplementary Table 1: Clinicopathological data of the study cohort

Primer for RT-qPCR		
**Name**	**Forward**	**Reverse**
RT_hRalA	ATGGCTGCAAATAAGCCCAAG	TGTCTGCTTTGGTAGGCTCATA
RT_hRalB	AGCCCCTGACGCTTCAGTTC	AGCGGTGTCCAGAATATCTATCT
RT_hNKIRAS1	GCAAGGTTGTGGTTTGTGGA	CTCCTCGGTCTGTTTCTACTG
RT_hNKIRAS2	TGTAGTGGGTTCGGAGATGAT	CGGTCTGTCTCAATGGAGCC
RT_mNKIRAS1	CTGCACTTGTATGACACCCGA	CCTAACACAACGATTGCCACC
RT_mNKIRAS2	GGAGCAGGTGCGTTTCTATGA	CGACTCTCGCCTGTCTGTGCTGTAGA
RT_mTNF	CTGGGACAGTGACCTGGACTGT	ACTCTCCCTTTGCAGAACTCAGG
RT_mIL-6	ATGAAGTTCCTCTCTGCAAGAGACTTCC	CCTGATTATATCCAGTTTGGTAGCATCC
RT_mIL-1b	ACCTTCCAGGATGAGGACATGA	CTAATGGGAACGTCACACACCA
RT_mArg1	CTCCAAGCCAAAGTCCTTAGAG	AGGAGCTGTCATTAGGGACATC
RT_mGAPDH	GGTGGAAGGTCGGTGTGAACG	CTCGCTCCTGGAAGATGGTG
RT_Actin	GCTGTGCTGTCCCTGTATGCCTCT	CCTCTCAGCTGTGGTGGTGAAGC
RT_mAmylase	CAAAATGGTTCTCCCAAGGA	ACATCTTCTCGCCATTCCAC
RT_mCK19	ACCCTCCCGAGATTACAACC	CAAGGCGTGTTCTGTCTCAA
RT_mInsulin	GGACCTTCAGACCTTGGC	GTTGCAGTAGTTCTCCAGCTGGTA
RT_rNKIRAS1	AGGTTGCAAGGTTGTCGTTT	CATGTACACGTCTTCCAGCG
RT_rNKIRAS2	CATCTGTGGGCAAAACGTCA	CCTCCTGGGTCTCGATCATC
RT_rSox9	GTGCTGAAGGGCTACGACTGGA	GTTGTGCAGATGCGGGTACTGG
RT_rHNF1b	TCCCATCTGCAATGGTGGTC	GCTGTGCACAAAGTGAGTGG
**Primers for end-point PCR**		
**Name**	**Forward**	**Reverse**
rkBRas1screen1	CAACAAGCATCTCCCACCAG	TGAACTCAGCCACACATCCT
rkBRas2screen1	GTTGTCCTGTCTCTGCCTCT	TTTCCTTCCCCTCCATTGCT
hkBRas1 screen	TGCCCACTGGAAAGTTATGT	TGTGCCTGGCCTGTATTCTT
hkBRas2 screen	CACAAAACACTGTTCCTGCTTAATT	CAAACAAACAAACAAAAGAAGGGCT
**Guide RNA sequences**		
**Name**	**Forward**	**Reverse**
rκB-Ras1 Guide1	CACCGACCGGATTCTTGCCCACTGT	AAACCAGTGGGCAAGAATCCGGTC
rκB-Ras1 Guide 2	CACCGATAGTGTGATTCCCATAAAG	AAACCTTTATGGGAATCACACTATC
rκB-Ras2 Guide 1	CACCGTCAAACCACGGGGTGGAGAT	AAACATCTCCACCCCGTGGTTTGAC
rκB-Ras2 Guide 2	CACCGACAACATGGTTCCCGTACAG	AAACCTGTACGGGAACCATGTTGTC
hκB-Ras1 Guide1	CACCGGTTGATGGTACATAAAGGGG	AAACCCCCTTTATGTACCATCAACC
hκB-Ras1 Guide 2	CACCGAATGCTTATTGCTCTAAGGG	AAACCCCTTAGAGCAATAAGCATTC
hκB-Ras2 Guide 1	CACCGTTGAAGAACAGAAGCACGCT	AAACAGCGTGCTTCTGTTCTTCAAC
hκB-Ras2 Guide 2	CACCGGGACCCCTTAAACCACCAAAG	AAACCTTTGTGGTTTAAGGGGTCCC
**shRNA Oligonucleotides**		
**Name**	**Forward**	**Reverse**
shRalA/B#1 (human)	CCGGGATAACTACTTTCGGAGTGGGCTCGAGCC CACTCCGAAAGTAGTTATCTTTTTG	AATTCAAAAAGATAACTACTTTCGGAGTGGGCTCGA GCCCACTCCGAAAGTAGTTATC
shRalA/B#3 (human)	CCGGGCTGGGCAAGAGGACTACGCACTCGAGT GCGTAGTCCTCTTGCCCAGCTTTTTG	AATTCAAAAAGCTGGGCAAGAGGACTACGCACTCG AGTGCGTAGTCCTCTTGCCCAGC
shRalA/B#1 (murine/rat)	CCGGGAGTTTGTAGAAGACTATGACTCGAGTCA TAGTCTTCTACAAACTCTTTTTG	AATTCAAAAAGAGTTTGTAGAAGACTATGACTCGA GTCATAGTCTTCTACAAACTC
shRalA/B#2 (murine/rat)	CCGGGACAACTACTTCCGAAGCGGACTCGAGTC CGCTTCGGAAGTAGTTGTCTTTTTG	AATTCAAAAAGACAACTACTTCCGAAGCGGACTCG AGTCCGCTTCGGAAGTAGTTGTC
shRalA/B#3 (murine)	CCGGGACTATGAGCCCACCAAAGCCTCGAGGCT TTGGTGGGCTCATAGTCTTTTTG	AATTCAAAAAGACTATGAGCCCACCAAAGCCTCGA GGCTTTGGTGGGCTCATAGTC
shmSox9#1	CCGGCGTCAATGAGTTTGACCAATACTCGAGTA TTGGTCAAACTCATTGTTTTTG	AATTCAAAAACGTCAATGAGTTTGACCAATACTCGA GTATTGGTCAAACTCATTG
shmSox9#2	CCGGGCGGAGGAAGTCGGTGAAGAACTCGAGT TCTTCACCGACTTCCTC CGCTTTTTG	AATTCAAAAAGCGGAGGAAGTCGGTGAAGAACTCG AGTTCTTCACCGACTTCCTCCGC
shmSox9#3	CCGGGCGACGTCATCTCCAACATTGCTCGAGCA ATGTTGGAGATGACGTCGCTTTTTG	AATTCAAAAAGCGACGTCATCTCCAACATTGCTCGA GCAATGTTGGAGATGACGTCGAC
shControl	CCGGCCTAAGGTTAAGTCGCCCTCGCTCGAGCG AGGGCGACTTAACCTTAGGTTTTTG	AATTCAAAAACCTAAGGTTAAGTCGCCCTCGCTCGA GCGAGGGCGACTTAACCTTAGGA
**Oligonucleotides for EMSA**		
**Name**	**Sequence**	
NF-κB	CAG GGC TGG GGA TTC CCC ATC TCC ACA GG	
Oct1	GAT CGT GTA TGC AAA TAA GGT C	
TNFp NF-κB	TG AGG TCC GTG AAT TCC CAG GCT	

Supplementary Table 2: Oligonucleotides
