# Supplementary material for: κB-Ras and Ral GTPases regulate acinar to ductal metaplasia during pancreatic adenocarcinoma development and pancreatitis
Source: Nat Commun. 2020 Jul 8;11:3409. doi: 10.1038/s41467-020-17226-0 (PMC7343838; doi:10.1038/s41467-020-17226-0)
Supplement: Supplementary file 3 — Reporting Summary [file 41467_2020_17226_MOESM3_ESM.pdf]

nature research

Corresponding author(s): Andrea Oeckinghaus

Last updated by author(s): Jun 8, 2020

Reporting Summary

Nature Research wishes to improve the reproducibility of the work that we publish. This form provides structure for consistency and transparency in reporting. For further information on Nature Research policies, see Authors & Referees and the Editorial Policy Checklist.

Statistics

For all statistical analyses, confirm that the following items are present in the figure legend, table legend, main text, or Methods section.

n/a	Confirmed	
☐	☒	The exact sample size (*n*) for each experimental group/condition, given as a discrete number and unit of measurement
☐	☒	A statement on whether measurements were taken from distinct samples or whether the same sample was measured repeatedly
☐	☒	The statistical test(s) used AND whether they are one- or two-sided<br>*Only common tests should be described solely by name; describe more complex techniques in the Methods section.*
☒	☐	A description of all covariates tested
☒	☐	A description of any assumptions or corrections, such as tests of normality and adjustment for multiple comparisons
☐	☒	A full description of the statistical parameters including central tendency (e.g. means) or other basic estimates (e.g. regression coefficient) AND variation (e.g. standard deviation) or associated estimates of uncertainty (e.g. confidence intervals)
☐	☒	For null hypothesis testing, the test statistic (e.g. *F*, *t*, *r*) with confidence intervals, effect sizes, degrees of freedom and *P* value noted<br>*Give P values as exact values whenever suitable.*
☒	☐	For Bayesian analysis, information on the choice of priors and Markov chain Monte Carlo settings
☒	☐	For hierarchical and complex designs, identification of the appropriate level for tests and full reporting of outcomes
☒	☐	Estimates of effect sizes (e.g. Cohen's *d*, Pearson's *r*), indicating how they were calculated

Our web collection on statistics for biologists contains articles on many of the points above.

Software and code

Policy information about availability of computer code

Data collection	Cytometry: BD FACSDiva version 6.1.3, BD FACSuite version 1.0.6<br>Immunoblot : Image Studio Software version 5.2 (LI-COR)<br>qPCR analysis: 7300 System SDS Software version 2.3
Data analysis	Cytometry: FlowJo version 10 (Treestar)<br>Immunoblot analysis: Image Studio Software version 5.2 (LI-COR)<br>qPCR analysis: 7300 System SDS Software version 2.3<br>Statistics: GraphPad Prism version 8<br>Immunofluorescence: Zeiss ZEN Black edition 2.3 SP1

For manuscripts utilizing custom algorithms or software that are central to the research but not yet described in published literature, software must be made available to editors/reviewers. We strongly encourage code deposition in a community repository (e.g. GitHub). See the Nature Research guidelines for submitting code & software for further information.

Data

Policy information about availability of data

All manuscripts must include a data availability statement. This statement should provide the following information, where applicable:

- Accession codes, unique identifiers, or web links for publicly available datasets
- A list of figures that have associated raw data
- A description of any restrictions on data availability

No datasets were generated or analysed during the current study. The data that support the findings of this study are available from the corresponding author upon reasonable request. The source data underlying Fig. 2 a, b, d, e, Fig. 3 h, i, Fig. 4 b, d-l, Fig. 5, Fig. 8 b-m and Supplementary Fig. 1 a, Fig. 3 c-h, Fig. 4 a, b, e-k, Fig. 5 a, d, Fig. 6c, Fig. 8 b, d-i are provided as a Source Data file.

Field-specific reporting

Please select the one below that is the best fit for your research. If you are not sure, read the appropriate sections before making your selection.

☒ Life sciences ☐ Behavioural & social sciences ☐ Ecological, evolutionary & environmental sciences

For a reference copy of the document with all sections, see nature.com/documents/nr-reporting-summary-flat.pdf

Life sciences study design

All studies must disclose on these points even when the disclosure is negative.

Sample size	All experiments were performed using cell lines, pimary cells or animals.<br>No statistical methods were used to predetermine sample sizes for experiments using cell lines or primary cells. A sample size of three was used as a starting point to evaulate the spread of the data (Casadevall A, Fang FC; Reproducible Science, Infect Immun, 2010 Dec; 78(12):4972-4975). Experiments were repeated more often if necessary to provide results with statistical significance.<br>For animal experiments sample sizes were estimated in advance where applicable based on experience- or literature-based knowledge on median values and standard deviations (t-test via "Power and Sample Size Calculation", MC Vanderbilt).
Data exclusions	No data were excluded from analysis.
Replication	All results reported were successfully replicated. We have conducted the experiments described in our paper at least twice, in most cases three or even more times.
Randomization	Animals of the same genotype were allocated to experimental groups randomly. Control animals were also assigned randomly as far as age- and sex-matching to the experimental group allowed.
Blinding	All histological analyses of the described phenotypes as well as rating of ADM scores were performed in a blinded fashion. Also cerulein injections were performed blinded towards the genotype of the animals. For all other experiments the investigators were not blinded. However, at least two investigators performed the reported experiments and analyzed the data independently.

Reporting for specific materials, systems and methods

We require information from authors about some types of materials, experimental systems and methods used in many studies. Here, indicate whether each material, system or method listed is relevant to your study. If you are not sure if a list item applies to your research, read the appropriate section before selecting a response.

Materials & experimental systems

n/a	Involved in the study
☐	☒ Antibodies
☐	☒ Eukaryotic cell lines
☒	☐ Palaeontology
☐	☒ Animals and other organisms
☐	☒ Human research participants
☒	☐ Clinical data

Methods

n/a	Involved in the study
☒	☐ ChIP-seq
☐	☒ Flow cytometry
☒	☐ MRI-based neuroimaging

Antibodies

Antibodies used	Antibodies from commercial sources:<br><br>anti-RalA: BD Biosciences, 610221, clone 8/ralA, Lot 3200797<br>anti-RalB: Origene, TA505880, clone OTI2C4, Lot W001<br>anti-Dab2: BD Biosciences, 610464, clone 52/p96<br>anti-α-tubulin: Santa Cruz, sc-23948, clone B-5-1-2, Lot D0414<br>anti-p53: Santa Cruz, sc-1312,clone M-19, Lot J1910<br>anti-Smad4: Santa Cruz, sc-7966, clone B8, Lot A2816<br>anti-phospho-p42/p44 MAPK: Cell Signaling, 9101, Thr202/Tyr204, Lot 27<br>anti-amylase1: Cell Signaling, D55H10<br>anti-phopsho-Akt Thr308: Cell Signaling, 4056, clone 244F9, Lot 13<br>anti-phospho-Akt Ser473: Cell Signaling, 4060, clone D9E<br>anti-phospho-Akt substrate: Cell Signaling, 9611<br>anti-Akt: Cell Signaling, 9272<br>anti-p44/p42 MAPK: Cell Signaling, 4695, clone 137F5<br>anti-Ras: Cell Signaling, 3965

anti-GAPDH: Proteintech, 60004-I-Ig
anti-NKIRAS2: Proteintech, 10174-1-AP
anti-phospho-Smad2: Cell Signaling, 3108T, clone 13804, Lot 8
anti-Smad2/3: Cell Signaling, 5678, clone D7G7, Lot 2
anti-Sox9: Cell Signaling, 82630, clone D8G8H, Lot 1
anti-phopsho-p65/RelA: Cell Signaling, 3033, Ser536, Lot 10
anti-Cpa1, Novus, AF2765, Lot W0D011808A
anti-phospho-TBK1: Cell Signaling, 5483, clone D52C2, Lot 8
anti-CD16/32: eBioscience, 101302, clone 93, Lot B195615
anti-CK19, Santa Cruz, sc-33111, clone D55410, Lot 1
anti-MIC1-1C3, Novus, NBP1-18961PE, Lot B-3-111317-PE
anti-Ki67: Cell Signaling, 12202, clone D3B5
IRDye®800CW anti- mouse: Licor, 926-32211
IRDye®680RD anti-rabbit: Licor, 926-68076
FITC goat anti-rat: BD Bioscience, 554016, polyclonal, Lot 6355831
Alexa594 goat anti rabbit: Thermo Fisher, A11012, polyclonal, Lot 1084427
PE/Cy7 anti CD45.2: Biolegend, 109829, clone 104
Alexa488 anti-rabbit: Thermo, A11008, Lot 1275894
Cy3 anti-goat: Jackson Immuno, 705-165-147
Cy3 anti-rabbit: Jackson Immuno, 711-165-152

Antibodies from non-commercial sources:

anti-kB-Ras: gift from Dr. S. Ghosh, Columbia University, NY, USA. Rabbit antibody raised against full-length human kB-Ras1
Mpx1 antibody: gift from Dr. C. Dorrell, specificity for surface staining of acinar cells provided in: Dorrell, C. et al. Isolation of mouse pancreatic alpha, beta, duct and acinar populations with cell surface markers. Mol. Cell. Endocrinol. 339, 144–150 (2011).

Validation

RalA, RalB and Dab2 antibodies were validated by testing on immunoblots with lysates from respective murine knock-down cell lines. Specificity of Sox9 antibody was tested via knockdown of Sox9 with three different shRNAs followed by IF analysis in murine pancreatic cells. Specificity of antibodies used to identify cell types in tissue sections was controlled by using two different antibodies for the same cell type.

The specificity of the non-commercial Mpx1 antibody was demonstrated by the donating lab using FACS sorting and respective RT-qPCR detecting known cell marker proteins in the sorted populations (primary murine pancreatic cells, Dorrell, C. et al. Isolation of mouse pancreatic alpha, beta, duct and acinar populations with cell surface markers. Mol. Cell. Endocrinol. 339, 144–150 (2011).). We have repeated this strategy to confirm specificity of the antibody aliquot provided to us.

The specificity of the kB-Ras antibodies used in this study in immunoblots was tested using both mouse and human knockout cells. For immunohistochemitry the antibody was tested on pancreatic tissue sections of kB-Ras knockout mice.

Validation data concerning all other antibodies purchased from commercial vendors are available on
the manufactures' websites and/or datasheets sent to us.

anti-α-tubulin: Santa Cruz, sc-23948, clone B-5-1-2, Lot D0414: Recommended for immunoblots of mouse and human cell lysates, validated for detecting murine and human endogenous protein.
anti-p53: Santa Cruz, sc-1312,clone M-19, Lot J1910: Recommended for immunoblots of mouse and human cell lysates, validated for detecting murine and human endogneous protein.
anti-Smad4: Santa Cruz, sc-7966, clone B8, Lot A2816: Recommended for immunoblot of mouse and human cell lysates, validated for detecting murine and human endogneous and exogenously expressed protein.
anti-phospho-p42/p44 MAPK: Cell Signaling, 9101, Thr202/Tyr204, Lot 27: Recommendend for IHC and immunoblots of mouse and human tissue and cell lysates, validated for detecting endogenous protein in stimulated mouse embryonic fibroblasts.
anti-amylase1: Cell Signaling, D55H10: Recommendend for IHC of mouse and human tissue, validated for staining of endogenous protein in human and rat pancreas.
anti-phopsho-Akt Thr308: Cell Signaling, 4056, clone 244F9, Lot 13: Recommendend for immunoblots of mouse and human cell lysates, validated for stimulated murine cells.
anti-phospho-Akt Ser473: Cell Signaling, 4060, clone D9E: Recommended for immunoblots and IHC of human and murine samples. Validated for staining of endogenous protein of human breast and lung carcinoma and PTEN heterozygous mouse endometrium.
anti-phospho-Akt substrate: Cell Signaling, 9611: recommended for IHC and immunoblot of human and murine samples. Validated for staining of human breast carcinoma by lambda phosphatase treatment.
anti-Akt: Cell Signaling, 9272. Recommended for IHC and immunoblot of human and murine samples. Validated for immunoblot in hamster and human cells using SignalSilence Akt siRNA. Validated for detecting endogenous protein of murine NIH/3T3 cells.
anti-p44/p42 MAPK: Cell Signaling, 4695, clone 137F5: Recommended for IHC and immunoblots of human and murine samples. Validated for detecting endogenous protein in human and murine cells using SignalSilence p44/42 MAPK siRNA. Validated for staining endogenous protein in human breast carcinoma by using a blocking peptide.
anti-Ras: Cell Signaling, 3965: Recommended for immunoblot of human and murine cell lysates. Validated for detecting endogenous protein in human, rat and murine cell lines.
anti-GAPDH: Proteintech, 60004-I-Ig: Recommended for immunoblots of mouse and human cell lysates, validated for detecting murine and human endogenous protein.
anti-phospho-Smad2: Cell Signaling, 3108T, clone 13804, Lot 8: recommended for immunoblot of human and murine cell lysates. Validated for detecting endogenous protein in human and murine cell lines, using TGFb treated cells as positive controls.
anti-Smad2/3: Cell Signaling, 5678, clone D7G7, Lot 2: recommended for immunoblot of human and murine cell lysates. Validated for detecting endogenous protein in human cells.
anti-phopsho-p65/RelA: Cell Signaling, 3033, Ser536, Lot 10: Recommended for flow cytometry analysis of murine cells. Validated to detect endogenous protein in murine and human cells, using TNF-alpha-treated cells as positive controls.

anti-Cpa1, Novus, AF2765, Lot W0D011808A: Recommended for IHC of murine tissue.
anti-phospho-TBK1: Cell Signaling, 5483, clone D52C2, Lot 8: Recommended for flow cytometry analysis of murine cells. Validated for detecting endogenous expression in human cells, using differentiated and undifferentiated THP-1 cells.
anti-CD16/32: eBioscience, 101302, clone 93, Lot B195615: recommended for flow cytometry of murine cells. Validated for staining murine splenocytes.
anti-CK19, Santa Cruz, sc-33111, clone D55410, Lot 1: Recommended for IHC of mouse and human tissue, validated for staining of endogenous protein in human kidney, breast, gall bladder.
anti-Ki67: Cell Signaling, 12202, clone D3B5: Recommended for IHC of mouse tissue, validated for staining of endogenous protein in murine colon and spleen.

Eukaryotic cell lines

Policy information about cell lines

Cell line source(s)	Panc1, MiaPaca2 and HEK293FT cells were obtained from ATCC and kindly provided by Dr. S. Ghosh. AR42J cells were purchased from CLS Cell Lines Service. PDC lines were generated as described in the manuscript.
Authentication	Generated PDC lines were characterized by RT-qPCR. Commercially obtained cell lines were authenticated by ATCC or CLS.
Mycoplasma contamination	Cell lines were not routinely tested for mycoplasma contamination.
Commonly misidentified lines (See ICLAC register)	No commonly misidentified cell lines were used.

Animals and other organisms

Policy information about studies involving animals; ARRIVE guidelines recommended for reporting animal research

Laboratory animals	As laboratory animals we used mice (Mus musculus). All mouse lines were on a C57BL/6J background. Animals of both sexes were used in experiments/analyses at an age range of 4 to 32 weeks. For the different experiments/analyses presented in this study the respective age of the animals used is indicated in the figure legends.
Wild animals	The study did not involve wild animals.
Field-collected samples	The study did not involve samples collected form the field.
Ethics oversight	All animal experiments were approved by the local authorities (Landesamt für Natur, Umwelt und Verbaucherschutz LANUV Nordrhein-Westfalen, Fachbereich 81) and the office of animal welfare of the University Clinic Münster.

Note that full information on the approval of the study protocol must also be provided in the manuscript.

Human research participants

Policy information about studies involving human research participants

Population characteristics	Detailed clinicopathological characteristics of the 61 consecutive PDAC patients who were included in the study were retrieved from the respective pathology reports/clinical records and are summarized in Supplementary Figure 1a. All patients were treated at the University Hospital Muenster Medical Center. Only patients with fully available clinical history as well as tissue samples of sufficient amount and quality were included in the study.
Recruitment	Patient samples were identified restrospectively from the archive of the Gerhard Domagk Institute of Pathology, University Hospital Muenster, according to the criteria stated above.
Ethics oversight	The use of human tissue samples was approved by the ethics committee of the University of Münster (Approval Number 2015-102-f-S).

Note that full information on the approval of the study protocol must also be provided in the manuscript.

Flow Cytometry

Plots

Confirm that:

☒ The axis labels state the marker and fluorochrome used (e.g. CD4-FITC).

☒ The axis scales are clearly visible. Include numbers along axes only for bottom left plot of group (a 'group' is an analysis of identical markers).

☒ All plots are contour plots with outliers or pseudocolor plots.

☒ A numerical value for number of cells or percentage (with statistics) is provided.

Methodology

Sample preparation	Murine pancreata were resected, minced into 2-4 mm small pieces in 5 ml cold HBSS, and washed 2 times with 10 ml of cold HBSS. Minced pancreata were digested in 30 mg/ml dispase I (Sigma), 30 mg/ml collagenase IV (Worthington-Biochem) supplemented with soybean trypsin inhibitor (Gibco, 0.1 mg/ml) and DNaseI (20 µg/ml) at 37°C for 60-80 min, carefully pipetting every 15 min. The single cell suspension was washed two times with cold RPMI without phenol red, supplemented with 10 % FCS. Cells were resuspended in RPMI containing 2 % FCS and filtered through a sterile 40 µm cell strainer. Cells were surface-stained with MIC1-1C3 (Novus) and/or Mpx1 antibodies (both 1:200, 15min, on ice, dark). For intracellular stainings, cells were additionally fixed and permeabilized using the FoxP3/Transcription Factor staining Buffer or Intracellular Fix & Perm Sets (eBioscience). Intracellular stainings were performed for 1h on ice. For quantification of leukocytes, cells were blocked with anti-CD16/32 (1:100, 10 min, ice, dark) and then stained with PE/Cy7 anti-mouse CD45.2 (Clone 104, Biolegend) for 30 min.
Instrument	Analysis of flow cytmetry experiments was performed on a FACS Aria III (BD Biosciences).
Software	BD FACSDiva software and BD FACSuite software were used for data collection and FlowJo version 10 (Treestar) for analysis of data.
Cell population abundance	Sorted cell populations were directly analyzed for expression of independent marker proteins by RT-qPCR without further determination of purity.
Gating strategy	Based on cell size (forward scatter, FSC) and cell granularity (sideward scatter, SSC), cell debris were excluded. Subsequently, FCS and SSC doublets, as well as dead cells defined as 7-AAD positive cells, were excluded. Cell debris, doublets and dead cell exclusion were applied in all flow cytometry analysis. For identification of metaplastic cells, acinar cells were additionally excluded via FSC/SSC gating.

☒ Tick this box to confirm that a figure exemplifying the gating strategy is provided in the Supplementary Information.
